# Modeling dosimetric benefits from daily adaptive RT for gynecological cancer patients with and without knowledge‐based dose prediction

**DOI:** 10.1002/acm2.14596

**Published:** 2025-01-27

**Authors:** Rupesh Ghimire, Lance Moore, Daniela Branco, Dominique L. Rash, Jyoti S. Mayadev, Xenia Ray

**Affiliations:** ^1^ Department of Radiation Medicine and Applied Sciences UC San Diego Health La Jolla California USA

**Keywords:** adaptive radiotherapy, cervical cancer, cone‐beam CT, dosimetry, IGRT, target margins

## Abstract

**Purpose:**

Daily online adaptive radiotherapy (ART) improves dose metrics for gynecological cancer patients, but the on‐treatment process is resource‐intensive requiring longer appointments and additional time from the entire adaptive team. To optimize resource allocation, we propose a model to identify high‐priority patients.

**Methods:**

For 49 retrospective cervical and endometrial cancer patients, we calculated two initial plans: the treated standard‐of‐care (Initial_SOC_) and a reduced margin initial plan (Initial_ART_) for adapting with the Ethos treatment planning system. Daily doses corresponding to standard and reduced margins (Daily_SOC_ and Daily_ART_) were determined by re‐segmenting the anatomy based on the treatment CBCT and calculating dose on a synthetic CT. These initial and daily doses were used to estimate the ART benefit (ΔDaily= Daily_SOC_‐Daily_ART_) versus initial plan differences (ΔInitial= Initial_SOC_–Initial_ART_) via multivariate linear regression. Dosimetric benefits were modeled with initial plan differences (ΔInitial) of $Bowel\;V_{40Gy}$ (cc), $Bladder\;D_{50\%}$ (Gy), and $Rectum\;D_{50\%}$ (Gy). Anatomy (intact uterus or post‐hysterectomy), DoseType (simultaneous integrated boost [SIB] vs. single dose), and/or prescription value. To establish a logistic model, we classified the top 10% in each metric as high‐benefit patients. We then built a logistic model to predict these patients from the previous predictors. Leave‐one‐out validation and ROC analysis were used to evaluate the accuracy. To improve the clinical efficiency of this predictive process, we also created knowledge‐based plans for the ΔInitial plans ($\Delta Initial_{RP}$) and repeated the analysis.

**Results:**

In both $\Delta Initial_{Orig}$ and $\Delta Initial_{RP}$ our multivariate analysis showed low *R* ^2^ values 0.34–0.52 versus 0.14–0.38. The most significant predictor in each multivariate model was the corresponding ∆Initial metric (e.g., ΔInitial Bowel (V40 Gy), *p* < 1e−05). In the logistic model, the metrics with the strongest correlation to the high‐benefit patients were $Bowel\;V_{40Gy}$ (cc), $Bladder\;D_{50\%}$ (Gy), DoseType, and SIBDose prescription. The models for original and knowledge‐based plans had an AUC of 0.85 versus 0.78. The sensitivity and specificity were 0.92/0.72 and 0.69/0.80, respectively.

**Conclusion:**

This methodology will allow clinics to prioritize patients for resource‐intensive daily online ART.

## 1 INTRODUCTION

Cervical cancer is the fourth most common cancer type in women. The American Cancer Society estimates there will be 13,820 new cases with 4,00360 fatalities from this disease each year.^1^ The most common treatment modality in the United States is external beam radiotherapy, which delivers a therapeutic radiation dose to the target. Intensity modulated radiation therapy uses inverse optimization to create highly patient‐specific plans that attempt to maximally spare nearby healthy tissues and organs. However, delivering these plans is complicated by the daily changes in organ anatomy (bowel loops, bladder, rectum, and sigmoid) in addition to the changing shape^2^ and position of the targets themselves.^3^, ^4^ To account for the target changes in position, a large Planning Target Volume (PTV) margin of 7–15 mm is added to the Clinical Target Volume (CTV).^5^, ^6^ This approach is designed to ensure the regions of the tumor always receive the prescription dose; however, studies have shown the cervix and uterus position can vary as much as 4–6 cm.^3^, ^7^, ^8^ Additionally, these large margins increase the volume of normal tissues that receive high doses and thus increase the risk of gastrointestinal (GI) toxicities and other side effects from treatment.^9^, ^10^

The introduction of daily online adaptive radiotherapy (ART)^11^ presents an opportunity to improve radiation treatment targeting precision. In daily ART, the treatment plan is re‐optimized based on daily changes in the position of the target and oddrgans at risk (OARs). Several studies have demonstrated that ART can improve the accuracy of radiation delivery to the tumor while limiting exposure to nearby healthy tissues, potentially lowering the risk of damage.^12^, ^13^, ^14^ The benefits to the OARs are further increased when daily ART is combined with reduced CTV‐to‐PTV margins. This strategy has been shown to allow substantial dose reductions for cervical cancer patients^6^, ^15^

However, there are certain difficulties in implementing ART. Firstly, ART is resource‐intensive,^16^, ^17^ as it requires more on‐treatment time at the machines as well as skilled human operators. Thus, it is not currently possible at most institutions to adapt every patient that may benefit. Secondly, not all patients benefit equally from ART. Prior research, including our own, has shown that even within a homogenous cohort, some patients have significant improvements in dose metrics with ART while others have limited benefits.^18^, ^19^ This variability in patient gains combined with the elongated treatment times highlights the importance of identifying the patients most likely to benefit from ART to optimize outcomes and allocate resources efficiently.

In response to these challenges, we sought in this study to develop a model that can predict the individual gynecological (GYN) patients with the highest dosimetric benefits from ART using information available prior to treatment start. We aim to provide clinicians with a valuable tool to screen patients for adaptive therapy, ultimately improving GYN cancer care and potentially patient outcomes.

## 2 METHODS

All data in this study were performed with the approval of our Institutional Review Board #200135. The study used retrospective planning CTs and on‐treatment Cone‐Beam CTs (CBCT) from 49 cervical and endometrial cancer patients previously treated at our institution with external beam radiation. We simulated doses from daily non‐adaptive and adaptive techniques to determine the dosimetric benefits from ART. We then built models to predict the dosimetric changes to dose volume histogram (DVH) metrics and for high‐benefit patient status using patient and plan features available at the time of initial planning. Further details on these steps are provided below.

### 2.1 Patient dataset

This research builds on a previously published dataset of 20 cervical cancer patients that were homogenous in prescription dose and target volumes.^18^ We added 29 additional GYN patients to this dataset that differed in prescription and target schemas from the initial cohort. Specifically, the initial cohort included only patients receiving 45 Gy in 25fx to the cervix, intact uterus, parametria, and pelvic nodal volume. In the expanded dataset, patients were included even if they had a hysterectomy, had primary prescriptions of 47.6 Gy in 28fx or 50.4 Gy in 28fx, and/or had a simultaneous integrated boost (SIB). Patients were identified by searching through our clinical database for the last 9 years for all GYN cancer patients by diagnosis code. Patients who had their paraaortic nodes included in the target volume were filtered out of the dataset as this portion of the target volume was not captured with on‐treatment CBCTs, and thus we could not simulate daily adaptive plans. For patients with multiple treatment courses, the first course was used. Patients who received prior irradiation to the pelvis were excluded. All patients had a simulation CT, approved structure set, clinically treated radiation therapy plan, and on‐treatment CBCT.

**FIGURE 1:**
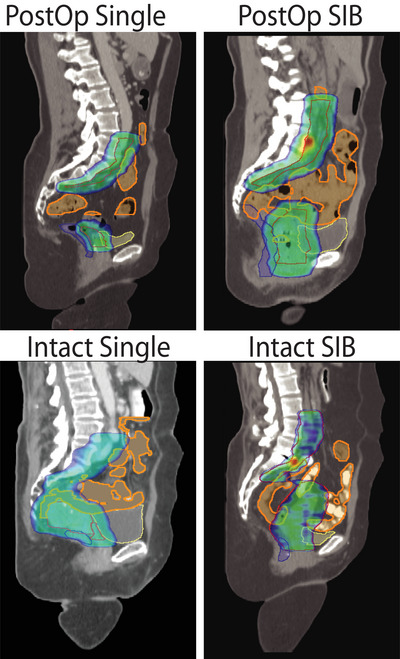
Patients were classified by target anatomy: PostOp if they had a hysterectomy or Intact if they had not, and by their prescription: SIB if they had a simultaneous integrated boost or Single for one dose level. Sagittal views for the cohorts are shown with dose clouds and OARs.

The resulting subset of 49 patients were categorized based on their target anatomy and prescription doses, Figure 1. First, patients were binarily categorized as either intact (if they had an intact uterus) or PostOp (if they had a hysterectomy prior to the radiation therapy). Subsequently, each of these groups was further divided into two subgroups: SIB and Single, depending on whether they received a SIB or single dose level to their PTV. This resulted in a total number of patients in each sub‐group of 20 Intact‐Single, nine Intact‐SIB, nine PostOp‐Single, and 11 PostOp‐SIB

Planning targets were drawn by physicians following consensus guidelines^5^ and included delineation of CTVs and internal target volume (ITV). The target compromised of cervix and uterus for patients with the uterus intact, parametria, and upper third of the vagina or upper half to two‐thirds of the vagina if it was clinically involved, and the pelvic nodes (common, external, internal, iliac, and presacral lymph nodes). Further, SIB group patients had additional gross tumor volumes (GTV) for the boost volume. Non‐adaptive plans used standard GYN CTV to PTV margins of 5–15 mm (5 mm on the nodal volume, 10 mm on the parametria, and 15 mm on the cervix–uterus ITV if intact or 1 mm on the vaginal–cervix if post‐op). The boosted nodes used 5 mm margins. The adaptive plans used a reduced CTV to PTV margin of 3 mm for all PTVs including the boosted nodes. Organs at risk on the planning CT included the bladder, rectum, sigmoid, and bowel‐bag. We added a bowel‐loops structure (called Bowel) that comprised the sigmoid, large bowel, and small bowel which corresponds to how the Ethos v1.1 auto‐segmentation software draws the bowel during adaptive sessions.

### 2.2 Data creation

**FIGURE 2:**
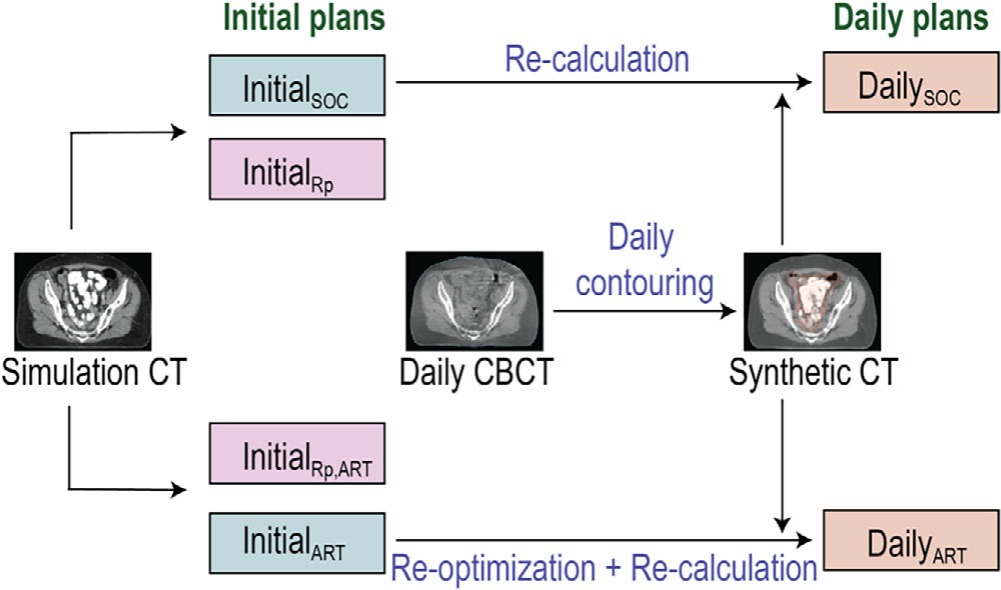
Flowchart of the creation of initial and daily plans from Sim and CBCT images. The initial plans are all calculated on the simulation CT image while the daily plans are calculated on a synthethic CT generated by the Ethos system using the contours from the Daily CBCT. Abbreviations: ART, adaptive radiotherapy plan with 3 mm margins; Rp, RapidPlan generated plan; SOC, standard of care plan with standard margins.

For the 49 patients, data metrics were extracted from six plans: for plans calculated on the simulation CT anatomy and two plans calculated on the representative daily anatomy. Figure 2 illustrates the workflow. First, the initial standard‐of‐care (SOC) plan ($Initial_{SOC\;}$) was obtained from our treatment database (Eclipse V16.0.0, Varian Medical Systems). This plan had been created manually, was used for treatment, and represented a high‐quality plan created using a non‐adaptive pathway with standard margins. Then a reduced 3 mm margin plan was created in the Ethos Treatment planning system ($Initial_{ART\;})$ as the reference plan for the daily adaptive plans. These plans were generated using an Ethos planning template as a starting point and then adding dose goals or adjusting their prioritization until a high‐quality plan was obtained. The default prioritized dose goals are available in the Supplementary Document for both the standard, (Figure S1) and SIB dose (Figure S2) templates. To improve OAR sparing, our in‐house RapidPlan models were also used with the templates; the Ethos TPS uses the predicted DVHs as line objectives for all matched OARs. Plan metrics were validated against the $Initial_{SOC\;}$ to ensure comparable clinical quality per patient in terms of dose coverage to targets, maximum dose, volume of 105%, and hot spots in OARs. OAR volume metrics were expected to improve due to the smaller volume and evaluated visually and against RapidPlan predicted DVHs to ensure appropriate sparing. Thus, this plan represented a high‐quality plan created using the adaptive pathway. Following our clinical practices, the $Initial_{SOC\;}$ plan used three arc VMAT, while $Initial_{ART\;}$ used nine field IMRT to reduce the on‐treatment re‐optimization time. In Eclipse, two more 3‐arc VMAT plans were created on the simulation CT using our clinical knowledge‐based planning model (RapidPlan, Varian Medical Systems, Palo, Alto)^13^, ^14^, ^15^ and either the SOC margin PTV from $Initial_{SOC\;}$ or the Adaptive Small margin PTV from $Initial_{ART\;}$, respectively. These plans ($Initial_{RP\;}$ and $Initial_{RP,ART\;})$ served as fast estimates of the dose metrics possible with either strategy and allowed us to evaluate if patient benefit could be predicted without fully optimizing both a non‐adaptive and adaptive reference plan prior to treatment. Thus, each RapidPlan was generated by optimizing with no manual user intervention beyond normalizing the final resulting plans to match the clinical plans (PTV V100% = 95%).

For the daily anatomy, an adaptive fraction was simulated for each patient using the last CBCT acquired during their treatment course and a Varian Ethos treatment emulator (version 1.1). The emulator uses an identical version of the registration, contouring, and treatment optimization software as the clinical treatment machine. During the simulated adaptive fraction, targets and OARs were edited based on the CBCT and a synthetic CT^17^ for dose calculation was auto‐generated by the system. The re‐optimized, reduced margin adaptive plan ($Daily_{ART}$) and structure set were exported to ARIA/Eclipse for metric extraction. In Eclipse, we registered the $Initial_{SOC\;}$ plan to the CBCT and recalculated the dose on the co‐registered synthetic CT to obtain the daily dose from the non‐adaptive pathway ($Daily_{SOC\;}$). Both the Ethos and Eclipse TPS use the same Acuros XB v16.01.0 algorithm and closed beam‐model for the Ethos Machine for dose calculation. Plans created on our other treatment machines used AAA v16.01.0, per our clinical practice. All plans used a single isocenter.

### 2.3 Dose metrics

From each of the above‐mentioned plans we extracted the $Bowel\;V_{40Gy}$ (cc), $Bladder\;D_{50\%}$ (Gy), and $RectumD_{50\%}$ (Gy). Bowel was contoured as individual bowel loops for this study. These metrics were chosen as they are part of our clinical standards and guidelines for plan evaluation from NRG GY006. Further, $Bowel\;V_{40Gy}$ (cc) was also found to be associated with GI toxicity of RTOG and LENTSOMA diarrhea symptoms.^20^ Similarly, dose‐effect relationship studies^21^, ^22^ have also shown that $Bladder\;D_{50\%}$ (Gy) and $Rectum\;D_{50\%}$ (Gy) are related to increased GI toxicity.

### 2.4 Predicted benefit and true adaptive benefit

The difference in each metric from margin reduction alone was calculated from the initial plans by subtracting $Initial_{ART\;}$ from $Initial_{SOC\;}$ for either the fully optimized plans or the RapidPlan‐generated plans, that is, $\Delta \;Initial_{Orig/Rp\;}=Initial_{SOC/Rp\;}\;-\;Initial_{ART/\;Rp,ART\;}$. The true adaptive benefit was calculated from the Daily plans by taking the difference of the $Daily_{SOC}$ and $Daily_{ART}$, that is, $\Delta Daily=Daily_{SOC\;}\;-\;Daily_{ART}$.

**FIGURE 3:**
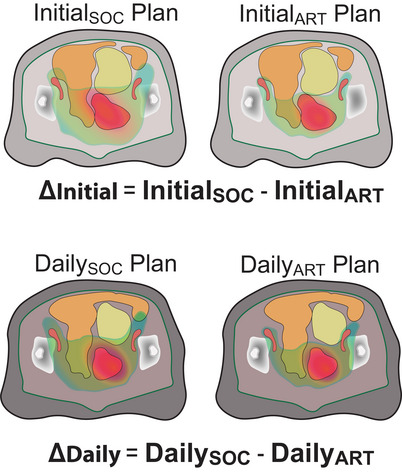
Illustration of the different plans. Initial metric differences (ΔInitial) are attributable to margin changes only. True adaptive benefits (ΔDaily) are dependent on reduced margins and re‐optimization for daily anatomy changes on CBCT. CTVs are shown in red, bladder in yellow, and bowel loops in orange.

Figure 3 shows the initial and the daily plans corresponding to the standard and the reduced margins. These differences were calculated for the dose metrics mentioned in Section 2.1 and were studied to subset the group of patients benefiting most from ART.

### 2.5 Linear regression models: Covariate selection and validation

We first aimed to predict the ΔDaily value for each metric using multivariate modelling. Included covariates included quantitative and categorical variables. The numerical variables were the ΔInitial value for the corresponding metric, the primary dose prescription, and the SIB dose prescription. Categorical variables were the dose type and the type of anatomy.

These variables were fit into a multivariate linear regression, Equation (1), using the glm() function of glmnet package^23^ in the R programming language to predict the Daily Adaptive benefit for the given structure.

(1) $$\begin{array}{cc} & \Delta Daily_{OARmetric}\;∼\;\Delta Initial_{OARmetric}+Anatomy\\ & \;+DoseType+PrimaryDoseRX+SIBDoseRX \end{array}$$

where,

$$\begin{array}{cc} OARmetric & =\mathrm{Either}\;\mathrm{Bowel}\;V40\mathrm{Gy},\;\mathrm{Rectum}\;D50\%,\\ & \;\;\mathrm{or}\;\mathrm{Bladder}\;D50\% \end{array}$$

Anatomy=IntactorPostOp

DoseTypes=SingleDoseorSIBDose

RX=PrescriptionDoseValue

A separate model was built to predict each of the three OAR metrics. We report the significance of the covariates in each model. Then we used the covariates with significance < 0.05 in a new model that we evaluated using leave‐one‐out cross validation (LOOCV). In this method, the model is fitted based on the entire cohort minus one left‐out patient. Then the model is used to generate a prediction for the left‐out patient. This process is repeated, leaving each patient out in turn. The predicted $\Delta Daily_{OARmetric}$ were compared against the true values using *R* ^2^ and Spearman's correlation coefficient. Both the model‐building and validation steps were then repeated replacing the $\Delta Initial_{Orig}$ covariates with $\Delta Intial_{RP}$ to evaluate if equivalent performance could be obtained without full clinical optimization of two plans.

### 2.6 Logistic model: Covariate selection and validation

We next designed a logistic model to identify patients likely to receive the greatest overall benefit. Patients were classified as high‐benefit if any one of their ΔDaily values was in the top 10% of our population's distribution of that metric. As we had 49 patients, this identified five patients for each metric, and the union of this group was classified as overall high‐benefit, resulting in a minimum of five patients (if there was overlap between metric groups) and a maximum of 15 (if there was no overlap). All remaining patients were classified as low‐benefit. We then used logistic regression with the glm() function of glmnet package^23^ in R to fit a model for the predicted probability of adapting as a function of the same covariates used in the previous quantitative model, Equation (2):

(2) $$\begin{array}{cc} & Probability\;of\;adapting\;∼\;\Delta Initial_{Bowel\;V40Gy}\\ & \;+\Delta Initial_{Bladder\;D50\%}+\Delta Initial_{RectumD50\%}\\ & \;+Anatomy+DoseType+PrimaryDoseRX\\ & \;+SIBDoseRX\; \end{array}$$

We identified the four strongest covariates via the alleffects () function of the effects package^24^ in *R*. This process for covariate selection was validated by also performing stepAIC regression using the step() function in the stats package^25^ in *R*.

To validate the predictive ability of the logistic model, we again used leave‐one‐out cross validation. Covariates included in each round were only the four strongest covariates identified from the initial logistic model. The predicted results were compared against the true classifications by performing Receiver Operating Curve (ROC) analysis. This required calculating the True Positive Ratio (TPR) and False Positive Ratio (FPR) for each classification threshold and then determining the total Area Under the Curve (AUC). We report the sensitivity, specificity, and threshold for adapting from this analysis We also calculated the sensitivity and specificity using the same threshold for each of the dose‐type cohorts (SIB vs. single‐dose).

Additionally, the entire covariate selection and logistic model validation was performed twice: once using the 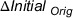 values in the model and once using the 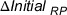 values in the model.

## 3 RESULTS

### 3.1 Changes in metrics on initial versus adaptive plans

**FIGURE 4:**
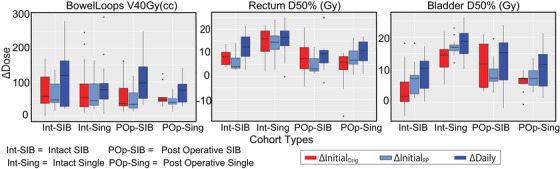
Boxplot of ∆Initial (Original and Rapidplan‐generated)P and ∆Daily for the metrics related to (a) Bowel Loops (b) Bladder and (c) Rectum across different cohorts. Note that there is substantial overlap in metric values between cohorts, suggesting that no entire group should be uniformly recommended for daily adaptation and instead, individual patients with any of the characteristics may have the most benefit from ART.

Figure 4 compares the changes in the initial versus daily plans, that is, (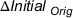, 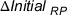 and ΔDaily). Changes in three DVH metrics are shown: $Bowel\;V_{40Gy}$ (cc), $Rectum\;D_{50\%}$ (Gy) and $Bladder\;D_{50\%}$ (Gy). These values are displayed for the four cohorts as given in the x‐axis: (a) intact SIB, (b) post operative SIB, (c) intact single, and (d) post operative single. The ΔDaily plan values had larger median changes compared to the changes in the initial plans. Additionally, there was overlap in the ΔDaily values from each cohort with no clear cohort‐based trends, suggesting the need of other statistical analysis techniques to find the highest benefitting patients.

### 3.2 Multivariate analysis

Table 1 shows the significance of each covariate in the multivariate models for the ΔDaily OAR metrics. Models were built first using values from only $\Delta Initial_{Orig\;}$ and then only $\Delta Initial_{Rp}$, with *p*‐values shown for both cases. The ΔInitial values for all three metrics $Bowel\;V_{40Gy}$ (cc), $Bladder\;D_{50\%}$ (Gy), and $Rectum\;D_{50\%}$ (Gy) showed high significance in predicting their respective ΔDaily using the Original value. Figure S3 plots these relationships for both the $\Delta Initial_{Orig\;}$ and $\Delta Initial_{RP\;}$ relationships. Additionally, dose type (SIB vs. Single) and dose prescription values were also moderately significant (*p* < 0.05) in the Bowel model. However, anatomy showed the least significance for all three Daily metrics. In general, metrics that were significant in the model when calculated from $\Delta Initial_{Orig\;}$ were less significant when calculated from $\Delta Initial_{Rp}$.

**TABLE 1:** The *p*‐values of and from the multivariate regression studies for bowel loops, bladder, and rectum.

$\Delta Daily_{OARmetric}$	Covariates	$\Delta Initial_{Orig\;}$	$\Delta Initial_{Rp\;}$
Bowel loops	$Bowel\;V_{40Gy}$	0E−7^***^	0E−5^***^
	Anatomy	0.374	0.858
	DoseType	0.002^*^	0.017
	PrimaryDoseRX	0.104	0.150
	SIBDoseRX	0.003^*^	0.021
Bladder	$Bladder\;D_{50\%}$ (Gy)	0.000^***^	0.087
	Anatomy	0.904	0.416
	DoseType	0.321	0.536
	PrimaryDoseRX	0.645	0.590
	SIBDoseRX	0.339	0.535
Rectum	$Rectum\;D_{50\%}$ (Gy)	0.000^***^	0.015
	Anatomy	0.620	0.802
	DoseType	0.187	0.396
	PrimaryDoseRX	0.036	0.004^*^
	SIBDoseRX	0.210	0.410

*notes*: The ΔInitial metric of the structure shows the greatest correlation compared to the other predictors. Significant Codes for *p*‐values: 0.0001 ‘***’ 0.001 ‘**’ 0.01 ‘*’.

Predictions were calculated for each patient using leave‐one‐out cross‐validation and a multivariate model with only the significant predictors from Table 1. The resulting *R* ^2^ values and Spearman's coefficient between the predicted and Actual $\Delta Daily_{OARmetric}$ are shown in Table 2. $\Delta Initial_{Orig\;}$ had slightly better *R* ^2^ values, 0.34–0.52 versus 0.14–0.38, in all cases compared to $\Delta Initial_{Rp}$ though all correlations were weak. The Spearman coefficients were also slightly better for the $\Delta Initial_{Orig\;}$ ranging from 0.6 to 0.66 suggesting a moderate monotonic relationship.

**TABLE 2:** *R* ^2^ and Spearman's coefficient values of the predicted metrics from the univariate model for each daily metrics.

	$\Delta Initial_{Orig\;}$		$\Delta Initial_{Rp\;}$	
$\Delta Daily_{OARmetric}$	*R* ^2^	Spearman	*R* ^2^	Spearman
Bowel loops	0.52	0.66	0.37	0.56
Bladder	0.34	0.60	0.14	0.37
Rectum	0.44	0.63	0.38	0.59

### 3.3 High‐priority patient classification

**FIGURE 5:**
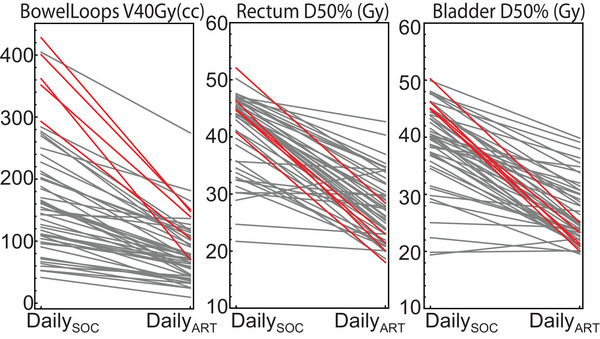
Metric decreases measured from the Daily SOC plans and Daily ART plans for each patient. Patients highlighted in red had decreases in top 10% for that metric. All patients with one or more red metric were classified as high‐benefit for logistic modeling.

Figure 5 shows the decrease in $Daily_{SOC\;}$ to $Daily_{ART\;}$ metrics for (a) $Bowel\;V_{40Gy}$ (cc), (b) $Bladder\;D_{50\%}$ (Gy), and (c) $Rectum\;D_{50\%}$ (Gy). These are the decreases from adapting with reduced margins. We classified the patients with ΔDaily values in the 90th percentile for each metric as high‐benefit, and these are highlighted in red. This resulted in 13 individual patients that were classified as high‐benefit in subsequent logistic modeling. We also observed that the patients with the largest decreases were not always the patients with the highest initial non‐adaptive values.

A few instances where metrics increased slightly with the smaller margins were also observed for the bladder and rectum. These increases were relatively small and evaluating them closer revealed they were an effect primarily of the IMRT adaptive plan allowing more of the low to medium dose to fall on one of the OARs (e.g., bladder) to achieve substantially better sparing of the other OAR (e.g., rectum) in‐turn. An example of this trade‐off with figures is included in Figure S4 to highlight this effect. Using VMAT planning for the adapted plans would likely allow for greater optimization of the low to medium isodose lines to negate this effect, but would also require additional time on treatment and thus could in‐turn require larger margins reducing this optimization benefit. It is also possible that using a larger number of IMRT fields could also improve this dosimetry without substantially increasing the optimization time though the delivery time will scale with increasing number of fields. In clinical practice, the Ethos planner may select the 12 field IMRT default arrangement or import a custom IMRT arrangement up to 19 fields to evaluate the impact on plan quality and determine the best tradeoff between these factors.

### 3.4 Logistic model: Covariate selection

**FIGURE 6:**
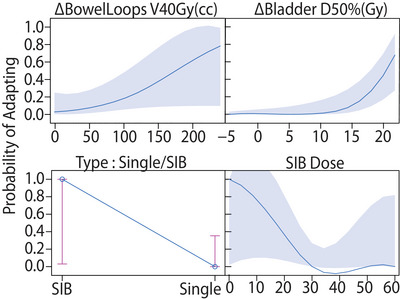
Curves representing the binomial model indicating the probability of adapting (y‐axis) for the four strongest covariates tested in the multivariate logistic model. The probability increases with increasing ∆Initial for Bowel and Bladder and with SIB status and dose. It should be noted that for SIB dose (bottom right plot) patients had values of either 0 (if receiving only single dose) or between 50 and 60 Gy. Thus while the curve is a continuous function, in the full model it either returns probability values of 1 or very close to 0.

In both the $\Delta Initial_{Orig}$ and $\Delta Initial_{Rp}$ logistic models, the metrics with the strongest correlation to the high‐benefit patients were $Bowel\;V_{40Gy}$ (cc), $Bladder\;D_{50\%}$ (Gy), DoseType, and SIBDose prescription. This process removed the terms containing rectum and anatomy information from the model. The reduced set of covariates was also consistent with the step AIC regression method for covariate selection. Figure 6 shows the individual effect of the four selected covariates on the predicted adaptive probability using the $\Delta Initial_{Orig\;}$ covariates.

This helped us reduce the number of covariates in Equation (1) to the form given below:

(3) $$\begin{array}{cc} & Probability\;of\;adapting\;∼\;\Delta Initial_{\mathrm{BowelV}40\mathrm{Gy}}\\ & \;+\Delta Initial_{\mathrm{BladderD}50\%}+DoseType+SIBDoseRX \end{array}$$

### 3.5 Logistic model: Validation

**FIGURE 7:**
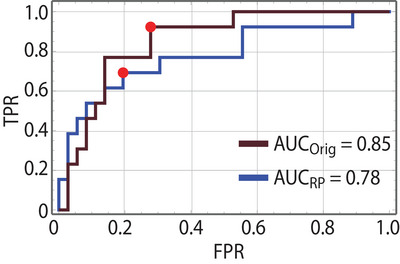
ROC curves for the logistic models using either 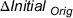 or 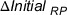 covariates. The AUCs are calculated and the threshold points are shown as red dots.

Figure 7 shows two ROC curves from the leave‐one‐out cross validation for the logistic models based on each initial plan type: 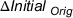 and 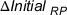.

The brown curve shows the ROC curve for the case of 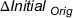 covariates. The AUC value is 0.85 and the red dot on the curve indicates the sensitivity and specificity values at the identified threshold. Results for the 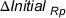 covariates are plotted in blue, which had a slightly lower AUC of 0.78.

Table 3 Presents the sensitivity and specificity for the full dataset for 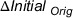 and 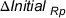, respectively. The sensitivity and specificity were also calculated for each dose type cohort to check if there was a bias in the full cohort for one or another type. The higher sensitivity in all the cohorts signify the model's accuracy to predict the patients who are likely to benefit from the cancer treatment regardless of dose regimen. While the moderately high specificity indicates that the majority of lower‐benefit patients are also identified correctly. Overall the model using 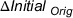 outperformed the model using 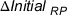 with higher sensitivity values although the specificity was slightly lower.

**TABLE 3:** Sensitivity and specificity values for different cohort types obtained using a fixed threshold from the ROC curves.

	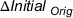		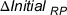	
Data	Sensitivity	Specificity	Sensitivity	Specificity
All patients	0.923	0.7222	0.6923	0.8056
SIB only	0.8333	0.7143	0.8333	0.9286
Single dose only	1.0000	0.7273	0.5714	0.7273

## 4 DISCUSSION

Daily online ART increases targeting precision and allows for reduction to normal tissues especially when combined with the use of reduced margins.^26^, ^27^, ^28^, ^29^, ^30^, ^31^, ^32^, ^33^ The clinical utilization of CBCT and MRI‐based daily adaptation has been growing quickly with the proliferation of commercial online adaptive platforms and the parallel publication of clinical trial results demonstrating improved outcomes.^34^, ^35^, ^36^, ^37^ However, our current clinical practice along with many published studies^38^, ^39^, ^40^, ^41^ have also shown the adaptive process is resource‐intensive due to the need for additional physician and physicist time at treatment and overall extended treatment times. Even on a high throughput machine such as the Varian Halcyon/Ethos, a standard appointment length of <12 min^42^ is effectively tripled with the adaptive workflow to 35–41 min^28^, ^40^ on average. Thus for effective clinical resource management, tools are needed to identify which patients are likely to receive the highest benefit within particular disease cohorts.

While there are models in the literature to predict the need for offline adaptation,^43^, ^44^, ^45^ little data exists for online ART. One previous study attempted to predict the need for pancreas SBRT online ART using patient characteristics (e.g., tumor size, prescription dose) and reference plan metrics but found neither to be strong predictors to adapt.^46^ We hypothesized that for GYN patients, the large margin reduction available with daily adaptive is the primary driver of the dosimetric benefit and thus the daily benefit may be predicted by generating two initials plans with standard‐of‐care versus reduced adaptive margins and using the difference in the metric as a predictor. This study built on our own previous work evaluating univariate models in a homogenous, single‐dose, intact 20‐patient cohort^18^ where we found the strongest predictor for the improvement in $Bowel\;V_{40Gy}$ (cc) were the difference metrics from the initial plans. In this study, we extended the patient dataset to include a greater variety of cervical and endometrial patients including cases that had hysterectomies as well as those being treated with SIB doses to their gross nodes.

We initially evaluated if knowing some basic parameters of the plan (intact/hysterectomy and SIB/Single Dose) was sufficient for identifying the highest benefit patients. This would be ideal as adaptive candidates could be identified prior to their simulation CT. However, our results (Figure 4) showed that high‐benefit patients could come from any combination of these two features, suggesting the need for a more nuanced method of identification.

We next classified high‐benefit patients in our group as those within the top 90th percentile on any single metric change (Figure 5). This high threshold was selected to force identification of only the highest benefit patients. We assumed most clinics would have limited capacity for adapting more than a few patients a day, and for instance at our clinic reserve only 2 h a day total for adaptive appointments out of a 10–12 h work day treating a total of 40–60 patients (6%–10% of treatments/day adapted). Of the total 15 highest benefitting patients in the daily plans of three structure metrics, we saw only two patients overlapped. Thus if a clinic valued high‐ and medium‐decreases in bowel V40 over high bladder and rectum decreases, they could classify patients differently while still using our overall approach to generate a model. We also observed that the highest‐benefit patients were not always those with the highest initial values for a metric on their SOC plan, suggesting a more nuanced look at the changes in the metrics and other plan parameters is necessary to predict high‐benefit status. When we built logistic models to predict these patients, we observed that the covariate selection resulted in only dose metrics for the $Bowel\;V_{40Gy}$ (cc) and $Bladder\;D_{50\%}$ (Gy) being used. Thus changes to rectum metrics seem to play a smaller role in overall adaptive benefit. This is likely due to the overall smaller variance in rectum D50% compared to the other metrics as seen in Figures 4 and 5. In general, rectum structures are more consistent across patients than bowel loops or bladders, and a similar amount is included in the target volume regardless of whether the patient has an intact uterus or large ITV.

From the results of the logistic models, we observed high sensitivity and specificity for the 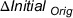 model. The 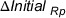 model had even higher specificity but lower sensitivity compared to the 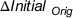. The AUC_orig_ (0.85) was also higher than the AUC_Rp_ (0.78) indicating superior performance when two high‐quality clinical plans are calculated: one conforming to standard clinical planning and another to clinical adaptive planning. However, using two Rapidplan generated plans still achieved strong performance and has substantial efficiency gains as the planner can run these automatically and a script can be written to provide the adaptive recommendation almost immediately. At that point, the planner could focus on iterating for a single high‐quality plan for whichever pathway was selected.

It should be noted that we only included OAR benefits as dosimetric benefits from adaptation, although improvements to target metrics could also be seen as motivation to adapt. This choice was motivated by our clinical experience with adaptation for cervical cancer patients where the largest benefits are seen to OAR metrics since daily plan reoptimization and renormalization ensures excellent target coverage. However, changes in maximum doses (e.g., D0.03cc) or conformity metrics (e.g., HI) can also occur and depending on clinical practice could take precedence over OAR changes. These metrics could be added into a new model, and should be especially considered if building a predictive model for other disease areas or SBRT regimens.

Even though the logistic model performance was high, the study has some shortcomings. Firstly, we assumed 3 mm adaptive margins were appropriate for all adaptive patients. These reduced margins have not yet proven to be suitable for routine clinical implementation in adaptive gyenecological cancer patients through a prospective study. Additionally, in clinical practice, patients experience some intrafraction motion during the online adaptive planning process and this could require larger patient‐specific margins. For instance, if one patient has challenges with bladder fill, they may need larger margins on the posterior/superior borders of the CTVp1 (uterus/cervix). Larger margins would decrease the OAR dose benefits modeled in this study, while using 3 mm margins in the presence of intrafraction motion could compromise target coverage. Future work could more precisely try to estimate the likelihood of large intrafraction motion on a patient‐specific basis, by using a metric measured from the change in CTVp1 position on the full and empty bladder ct simulation imageset. However, this is outside the scope of this study as we did not have repeat on‐treatment CBCT imaging data separated by the intervals likely to be seen for an adaptive treatment (e.g., 10–20+ min) which would be needed to validate such a metric. In lieu of this, we opted to predict the maximum benefit possible for each given patient. Correlating these benefits with on‐treatment benefits despite inter‐fraction motion is a necessary first step in predicting patient benefit from adaptation, and future work can evaluate incorporating more metrics to more precisely predict on‐treatment realized benefit of adaptation.

Another weakness of this study, is that we only considered one adaptive fraction while modeling the data, due to the substantial time it takes to create simulated daily fractions. In our previous study we observed that for the majority of patients there was a small standard deviation in the changes to adaptive metrics across five simulated weekly adaptive fractions compared to the variation across patients or the change in metrics from standard margins to adaptive margins.^18^ Thus, had we used an average from five simulated fractions across the treatment course it is likely that the predicted decrease from adaptive would have been slightly smaller for all patients, but that the overall classification of patients as high or low benefit would be similar. In this study, we used the patient's last CBCT and minimal 3 mm margins in order to approximate the absolute maximum adaptive benefit a patient may have. While a slightly larger margin schema would have also decreased these benefits, the effect is likely to be uniform across all patients and thus is expected to have minimal impact on the overall covariate selection and modeling performance. Building the model on a larger dataset likely has the potential to produce more generalizable results. Another potential weakness is that our model is built using simulated adaptive data rather than prospectively acquired adapted data. This is due to limitations in the number of adapted patients we have treated thus far and the inherent bias that is used to pre‐select prospectively adapted patients. We sought instead to build a model using as many different types of cervical/endometrial patients as we see in our clinic with the assumption that anyone may have a large adaptive benefit. There was also a difference in dose algorithms used for the standard margin initial and daily plans compared to the Ethos initial and adapted plans for some patients. This affected patients whose clinical initial plans were treated on a machine other than our Ethos, and thus used a AAA algorithm for Initial_SOC_ and Daily_SOC_. Because the bulk of our analysis deals with differences in SOC margin versus 3 mm margin plans, this impact of algorithm while suboptimal was consistent within their correlated data (e.g., 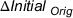 is the difference between a AAA plan with standard magins and an Acuros plan with 3  mm margins and ΔDaily is the difference between a AAA plan with standard magins and an Acuros plan with 3 mm margins for a specific patient). Lastly we validated our model using LOOCV which can risk over‐fitting as the dataset is small. Future work to expand the analysis dataset and use an independent validation cohort would be necessary prior to using the model in clinical practice.

This study could be expanded by modeling the impact of different choices of margin reduction on adaptive benefit. In this situation, the choice of margin would be its own variable within the model. This would require including a continuous range of plans with different margin reductions for the initial model‐fitting and was outside the scope of the current work but would allow for greater flexibility in clinical decision‐making.

Our current selection method for adaptive treatment is guided by our physicians'desire to adapt a particular patient cohort (e.g., all cervical cancer patients) and is limited by whether they think a patient will be able to tolerate the longer appointments and if our machine has availability for a 30+ min treatment. Currently this decision is thus often made at the time of consult or simulation, prior to reviewing the images and evaluating dose benefits or likelihood of internal daily motion. The physician's decision to recommend a patient for adaptive treatment care could be supplemented by a predictive model such as ours, which would be useful in managing clinical resources and ensure that we are providing all patients equal opportunity to this impactful technology. While we focused on GYN cancers, this modeling approach can be transferred to other patient disease sites

## 5 CONCLUSION

Our work aimed to predict the patients with the greatest dosimetric benefit from online ART treatment based on only their initial planning data. A logistic model that predicted the patients with DVH metric decreases in the top 90% of the cohort had an AUC of 0.85 if clinical‐quality initial plans were generated to provide the input covariate metrics. If knowledge‐based planning methods were used to generate the input covariates, a moderately high AUC of 0.78 was achieved. While the AUC was slightly decreased with the use of knowledge‐based planning, the overall planning efficiency is substantially increased and this method would likely be preferred clinically to provide a fast determination of whether a patient should be adapted. Thus by using initial planning metrics, our models can help clinicians quickly and efficiently identify which patients would most likely benefit from the adaptive planning.

## AUTHOR CONTRIBUTIONS

Rupesh Ghimire: generated and analyzed the data, contributed to the evaluation of the results, and wrote the manuscript. Lance Moore: contributed to data analysis, helped with scientific discussions, and revised the manuscript. Daniela Branco: contributed to the conception of this work, generated data, helped with scientific discussions, and revised the manuscript. Dominique Rash: reviewed and edited the contours, contributed to scientific discussions, and revised the manuscript. Jyoti S. Mayadev: reviewed and edited the contours, contributed to scientific discussions, and revised the manuscript. Xenia Ray: conceived the work, contributed to the evaluation of the results, and contributed to writing the manuscript. All authors reviewed the results and approved the final version of the manuscript.

## CONFLICT OF INTEREST STATEMENT

Dr. Ray is the recipient of honorariums and consulting fees from Varian Medical Systems and has a research agreement with Varian Medical Systems. She is also the recipient of consulting fees from KoRTUC. Dr. Mayadev is the recipient of consulting and honorarium fees from Merck, AstraZeneca, Primmune, KoRTUC, Agenus Bio, and Varian Medical Systems. Dr. Mayadev also discloses grant funding from ECTCN‐NCI, NRG Oncology, and NCI.

## Supporting information



Supporting Information
