# Supplementary material for: Modeling dosimetric benefits from daily adaptive RT for gynecological cancer patients with and without knowledge‐based dose prediction
Source: J Appl Clin Med Phys. 2025 Jan 27;26(3):e14596. doi: 10.1002/acm2.14596 (PMC11905257; doi:10.1002/acm2.14596)

SUPPLEMENTARY MATERIALS

Supplementary Figure 1: The default list of prioritized goals used to generate the Initial$_{ART, Ethos}$ single dose level plans. Note this list was adjusted as needed on a patient-specific level to ensure high plan quality similar to their Initial$_{SOC, Orig}$ plan. Adjustments including reprioritizing goals, adding additional goals, and lowering dose goals on OARs.

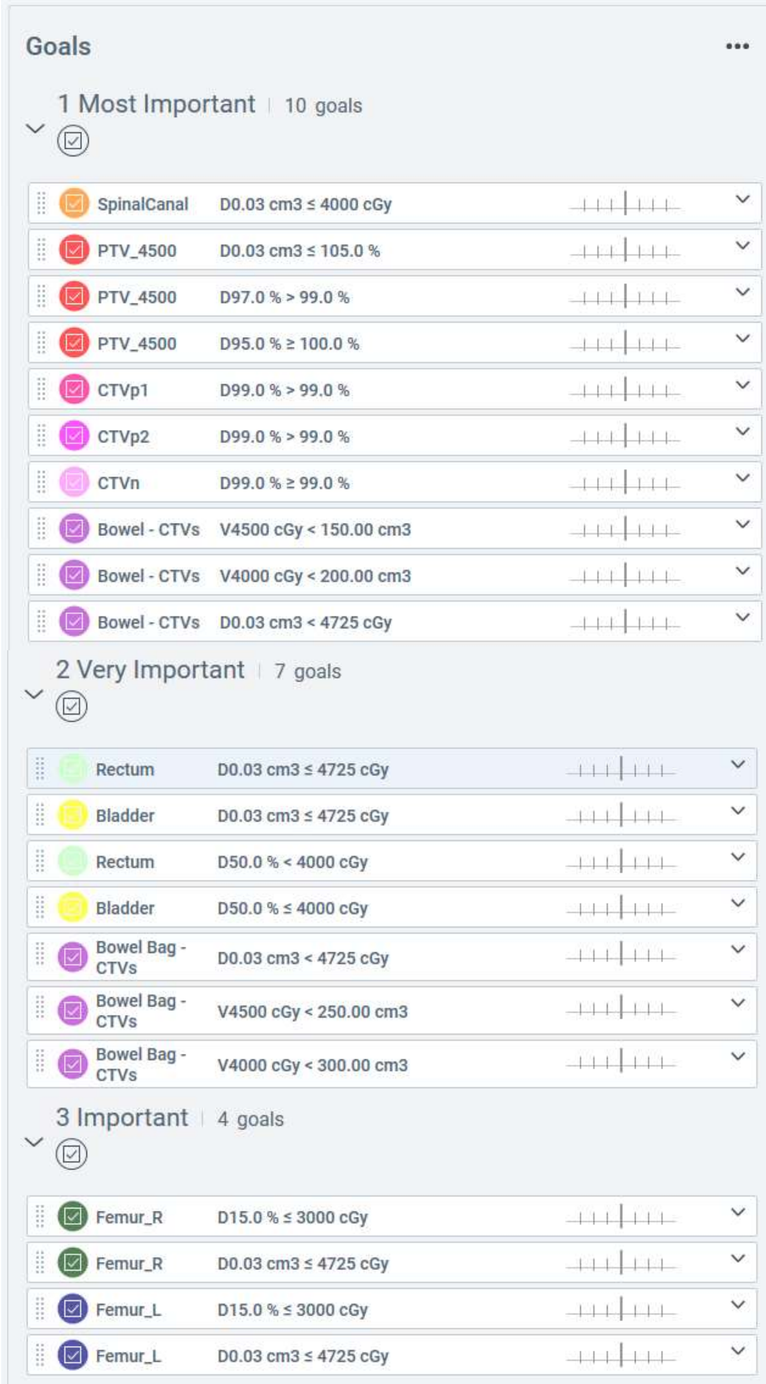

Supplementary Figure 2: The default list of prioritized goals used to generate the Initial$_{ART,\ Ethos}$ Simultaneous Integrated Boost plans. Note this list was adjusted as needed on a patient-specific level to ensure high plan quality similar to their Initial$_{SOC,\ Orig}$ plan. Adjustments including reprioritizing goals, adding additional goals, lowering dose goals on OARs.

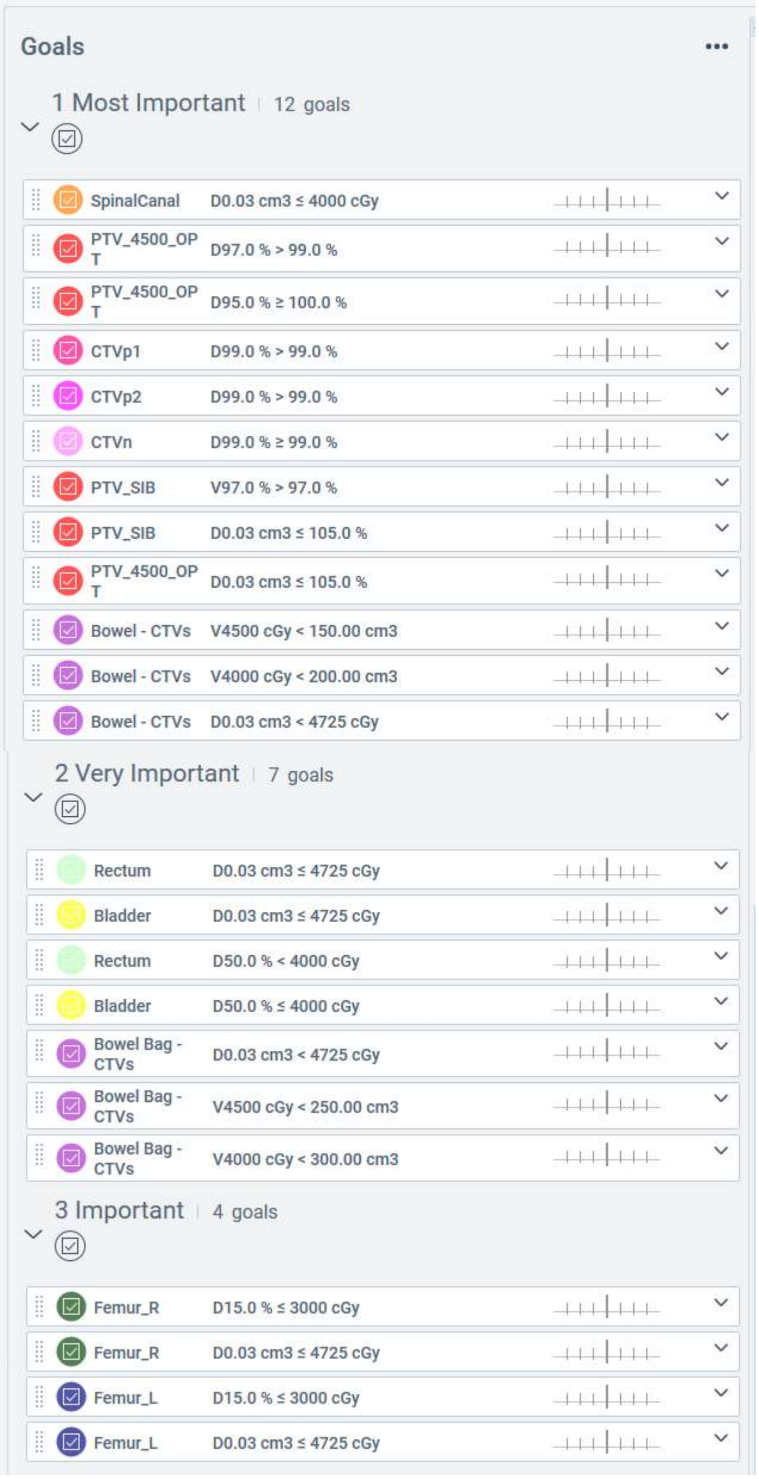

Supplementary Figure 3: The values for ΔDaily versus $\Delta InitialOrig$ or $\Delta InitialRp$ are shown for the 3 OAR Metrics.

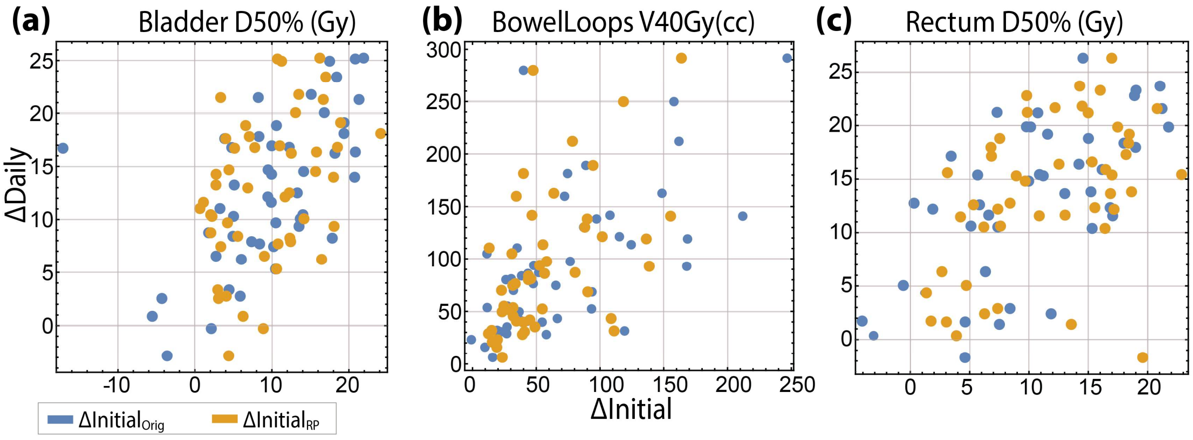

Supplementary Figure 4: Example of a patient where the DailySOC plan (left) had a lower Bladder D50% compared to the DailyART plan (right). The DVH comparison is also shown where Squares are the DailySOC and Triangles are the DailyART plans respectively. Note at high doses, DailyART allows for more bladder sparing, but the low to medium dose is higher. This is due to a trade-off for superior rectum sparing with the DailyART plan, which creates a much sharper gradient posteriorly. In the standard margin plan, almost the entire rectum overlaps with the PTV_4500 and thus there is little incentive to decrease the dose posteriorly. It is possible that using VMAT optimization for the adapted plan would allow for further sparing on the bladder even with this anatomical geometry.

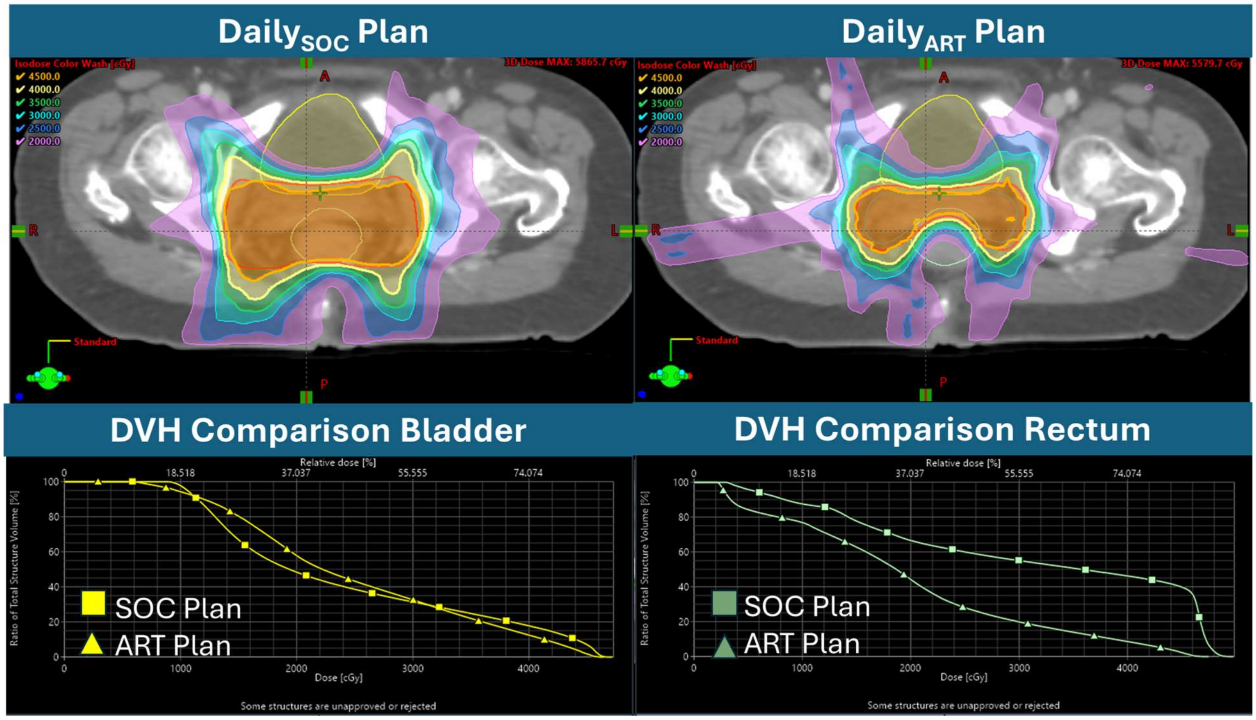

Supplement: Supplementary file 1 — Supporting Information [file ACM2-26-e14596-s001.pdf]
